# Supplementary material for: Metabolic engineering considerations for the heterologous expression of xylose-catabolic pathways in Saccharomyces cerevisiae
Source: PLoS One. 2020 Jul 27;15(7):e0236294. doi: 10.1371/journal.pone.0236294 (PMC7384654; doi:10.1371/journal.pone.0236294)
Supplement: S1 Text — (DOCX) [file pone.0236294.s001.docx]

**S1 Text. Supplementary Materials and Methods**

**Plasmid preparation**

Plasmids were constructed as previously described [[1](#_ENREF_1),[2](#_ENREF_2)]. The primers used in this study were synthesized by Cosmogenetech co. (Seoul, Korea) (S2 Table). Guide RNAs (gRNAs) with different sequences for the recognition of the target genes were designed as described in S2 Table. The pRS42H-PHO13.1 plasmid was constructed by modifying the previously described method [[3](#_ENREF_3)]. The gRNA cassette with sequence for the recognition of *PHO13* was synthesized by IDT (Coralville, IA, USA). Next, the gBlock-PHO13 cassette from pRS42H-PHO13.1 plasmid was digested by *Bam*HI and *Sal*I, and then ligated with pRS42K plasmid digested with the same enzyme. The resulting pRS42K-PHO13.1 plasmid was selected on the LBK agar plate and confirmed by *Xho*I. Other gRNA plasmids were constructed using the fast cloning method [[4](#_ENREF_4)]. Briefly, linearized pRS42H-ALD6.1, pRS42H-TAL1.1, pRS42H-SOR1.1, pRS42H-INT#1, pRS42H-INT#6, and pRS42H-INT#9 plasmids were PCR-amplified from the pRS42H-GND1.1 gRNA plasmids [[3](#_ENREF_3)] as a template DNA with Kim044/Kim045, Kim444/Kim445, Kim236/Kim237, Kim141/Kim142, Kim314/Kim315, and Kim498/Kim499 primers, respectively. Methylated PCR products by treatment with *Dpn*I was transformed into *E. coli*, which was selected on the LBA agar plate. Finally, the target sequence substitution was confirmed by sanger sequencing with the universal T3 primer (CAATTAACCCTCACTAAA).

The *xylA* gene derived from *Orpinomyces* sp. with codons optimized for *S. cerevisiae* was synthesized (GenBank No. MK335957). The *xylA* gene was then ligated with a pRS42H vector that had been linearized and dephosphorylated by treatment with *EcoR*V and CIP. The resulting plasmid, pRS42H-XI, was used as a template for the amplification of the *xylA* gene.

For overexpression of the *xylA* gene by delta integration method, the *xylA* cassette was PCR-amplified from pRS42H-XI plasmid as a template DNA with Kim018/Kim509 and digested by *Hind*III, and then ligated with pYS10 plasmid [[5](#_ENREF_5)] digested with the same enzyme. The resulting pYS-δXI plasmid was selected on the LBA agar plate and confirmed by *Hpa*I.

**Strain construction by Cas9-based genome integration**

Yeast transformation was used lithium acetate/single-stranded carrier DNA-polyethylene glycol method [[6](#_ENREF_6)]. CRISPR/Cas9 engineering technology was performed with some modifications of previously published methods [[1](#_ENREF_1),[2](#_ENREF_2),[3](#_ENREF_3)]. First, the laboratory *S. cerevisiae* D452-2 strain was introduced with the plasmid pRS41N-Cas9 and selected on YPD agar plate containing 100 μg/mL nourseothricin sulfate (Gold Biotechnology, St. Louis, USA). Second, to replace the *ALD6* gene with two isogenic xylose pathway genes, double-stranded repair DNAs (Donor DNAs) were amplified by PCR using Kim046/Kim047 primers. pSR6-X123 and pRS42H-XI plasmids were used as template DNAs for an oxidoreductase pathway- and an isomerase pathway-related donor DNA, respectively. The resulting donor DNAs were named Donor_ald6_XYL12, and Donor_ald6_xylA, respectively. Third, to construct the xylose-fermenting strains, the Cas9-expressing *S. cerevisiae* D452-2 strain was transformed with the pRS42H-ALD6.1 gRNA plasmid and either Donor_ald6_XYL12 or the Donor_ald6_xylA, and selected on YPD agar plate containing 100 μg/mL nourseothricin sulfate, and 300 μg/mL hygromycin B. gRNA plasmid (~ 4 μg) and Donor DNA(~ 4 μg) were transformed into the target strain. The resulting strains, the XYL12 and XI strains, were confirmed by colony PCR using Kim049/Kim078 primers. Fourth, to confirm the effect of *PHO13* disruption on the xylose pathway, *XYL3* gene-related donor DNAs were PCR-amplified from the pSR6-X123 plasmid using Kim068/Kim069 or Kim129/Kim130 primers, and named Donor_pho13_XYL3 and Donor_int#1_XYL3, respectively. Fifth, following the same procedure as above, the pRS42K-PHO13.1 gRNA plasmid and the Donor_pho13_XYL3 were transformed into the XYL12 and XI strains, respectively, and selected on YPD agar plate containing 100 μg/mL nourseothricin sulfate, and 300 μg/mL G418 sulfate. For control strains, the XYL12 and XI strains were transformed with the pRS42H-INT#1 gRNA plasmid and the Donor_int#1_XYL3, and selected on YPD agar plate containing 100 μg/mL nourseothricin sulfate and 300 μg/mL hygromycin B. Several colonies were confirmed by colony PCR using the following primers: Kim048/Kim077 (*pho13::XYL3*) and Kim143/Kim144 (*int#1::XYL3*). The resulting strains were named the XYL123, XI-XYL3, XYL123 *pho13*∆, and XI-XYL3 *pho13*∆ strains.

**Gene overexpression or deletion in the XI-XYL3 strain**

The individual gene overexpression (*xylA*, *XYL3*, and *TAL1*) or deletion (*sor1*∆) in the XI-XYL3 strain was performed by the CRISPR/Cas9 engineering technology as described above.

For the overexpression of *xylA*, the XI-XYL3 strain expressing the pRS41N-Cas9 plasmid was co-transformed with the pRS42H-INT#6 gRNA plasmid and the donor DNA generated from the pRS42H-XI plasmid by the Kim320/Kim321 primers, and yielding the (XI)_2_-XYL3 strain. Overexpression of *xylA* in the XI-XYL3 *pho13*∆ strain was also performed as described above. For the overexpression of *XYL3*, the XI-XYL3 strain expressing the pRS41N-Cas9 plasmid was co-transformed with the pRS42H-INT#9 gRNA plasmid and the donor DNA generated from the pRS42H-XYL3 plasmid by the Kim500/Kim501 primers, and yielding the XI-(XYL3)_2_ strain. For the clean deletion of *SOR1* (*sor1*∆), the XI-XYL3 strain expressing the pRS41N-Cas9 plasmid was co-transformed with the pRS42H-SOR1.1 gRNA plasmid and the donor DNA generated by the Kim238/Kim239 primers, and yielding the XI-XYL3 *TAL1* strain. The overexpression of *TAL1* in the XI-XYL3 strain was performed by the method described previously [[7](#_ENREF_7)]. All transforments were selected on YPD agar plate containing 100 μg/mL nourseothricin sulfate and 300 μg/mL hygromycin B, and several colonies were confirmed by colony PCR using the following primers: Kim326/Kim327 (*int#6::xylA*), Kim502/Kim503 (*int#9::XYL3*), Kim242/Kim243 (*sor1*∆), and SOO626/SOO676 (*TEF2*_P_*-TAL1*).

To construct the *gre3*∆ mutant, the *gre3::KanMX4* cassette was PCR-amplified from the genomic DNA of the BY4741 *gre3::KanMX4* strain of the Yeast Knockout Collection (Open Biosystems) by the Kim262/Kim263 primers. The PCR fragment was integrated to the XI-XYL3 strain, and then the resulting XI-XYL3 *gre3*∆ strain was selected on YPD agar plate containing 300 μg/mL G418 sulfate.

**Overexpression of *xylA* in the XI-XYL3 strain by δ integration method**

To overexpression of *xylA* gene in the XI-XYL3 strain, linearized pYS-δXI plasmid by *Kas*I was integrated to the XI-XYL3 strain, and then the resulting the δ(XI)-XYL3 strains were selected on SC-Leu agar plates (synthetic complete media; 6.7 g/L yeast nitrogen base with ammonium sulfate, 0.79 g/L of complete supplement mixture without leucine) containing 20 g/L glucose. The transformants were selected in YP medium containing 40 g/L xylose under oxygen-limited conditions (80 rpm) with a starting OD_600_ of 1.0.

**Deletion of *PHO13* in the δ(XI)-XYL3 strain**

The *PHO13* gene deletion in the δ(XI)-XYL3 strain was performed by the CRISPR/Cas9 engineering technology as described above. For the deletion of *PHO13*, the δ(XI)-XYL3 strain expressing the pRS41N-Cas9 plasmid was co-transformed with the pRS42H-PHO13.1 gRNA plasmid and the donor DNA generated by the Kim785/Kim786 primers, and yielding the δ(XI)-XYL3 *pho13*∆ strain. Transforment was selected on YPD agar plate containing 100 μg/mL nourseothricin sulfate and 300 μg/mL hygromycin B, and several colonies were confirmed by colony PCR by the Kim048/Kim077.

**References**

1. Ye S, Jeong D, Shon JC, Liu K-H, Kim KH, et al. (2019) Deletion of *PHO13* improves aerobic l-arabinose fermentation in engineered *Saccharomyces cerevisiae*. Journal of Industrial Microbiology & Biotechnology 46: 1725-1731.

2. Jeong D, Ye S, Park H, Kim SR (2020) Simultaneous fermentation of galacturonic acid and five-carbon sugars by engineered *Saccharomyces cerevisiae*. Bioresource Technology 295: 122259.

3. Kim SR, Xu H, Lesmana A, Kuzmanovic U, Au M, et al. (2015) Deletion of *PHO13*, encoding haloacid dehalogenase type IIA phosphatase, results in upregulation of the pentose phosphate pathway in *Saccharomyces cerevisiae*. Applied and Environmental Microbiology 81: 1601-1609.

4. Li C, Wen A, Shen B, Lu J, Huang Y, et al. (2011) FastCloning: a highly simplified, purification-free, sequence-and ligation-independent PCR cloning method. BMC Biotechnology 11: 92.

5. Jin Y-S, Jeffries TW (2003) Changing flux of xylose metabolites by altering expression of xylose reductase and xylitol dehydrogenase in recombinant *Saccharomyces cerevisiae*. Biotechnology for Fuels and Chemicals: Springer. pp. 277-285.

6. Gietz RD, Schiestl RH (2007) High-efficiency yeast transformation using the LiAc/SS carrier DNA/PEG method. Nature protocols 2: 31-34.

7. Xu H, Kim S, Sorek H, Lee Y, Jeong D, et al. (2016) *PHO13* deletion-induced transcriptional activation prevents sedoheptulose accumulation during xylose metabolism in engineered *Saccharomyces cerevisiae*. Metabolic Engineering 34: 88-96.
