# Supplementary figures and images for: Metabolic engineering considerations for the heterologous expression of xylose-catabolic pathways in Saccharomyces cerevisiae
Source: PLoS One. 2020 Jul 27;15(7):e0236294. doi: 10.1371/journal.pone.0236294 (PMC7384654; doi:10.1371/journal.pone.0236294)

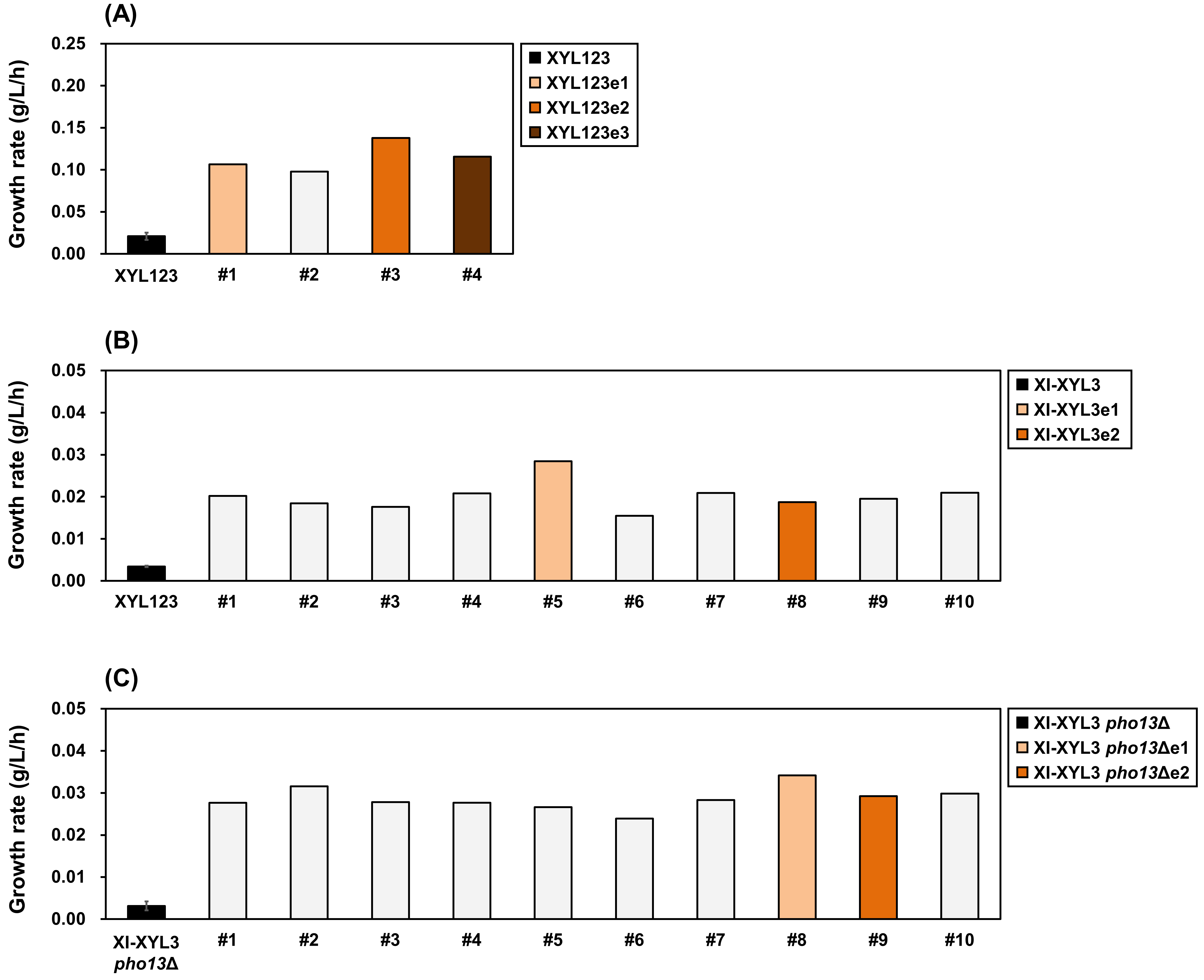

Supplement: S1 Fig — Growth rate comparison of the evolved colonies of the XYL123 (A), XI-XYL3 (B), and XI-XYL3 pho13Δ (C) strains. Two-three most promising colonies were selected from each group, and denoted to XYL123e, XI-XYL3e, and XI-XYL3 pho13Δe, respectively. Strains were cultured in YP medium containing either 40 g/L xylose (A) or 100 g/L xylose (B, C) under oxygen-limited conditions (80 rpm). Volumetric growth rates were calculated at the exponential phase. (TIF) [file pone.0236294.s002.tif]

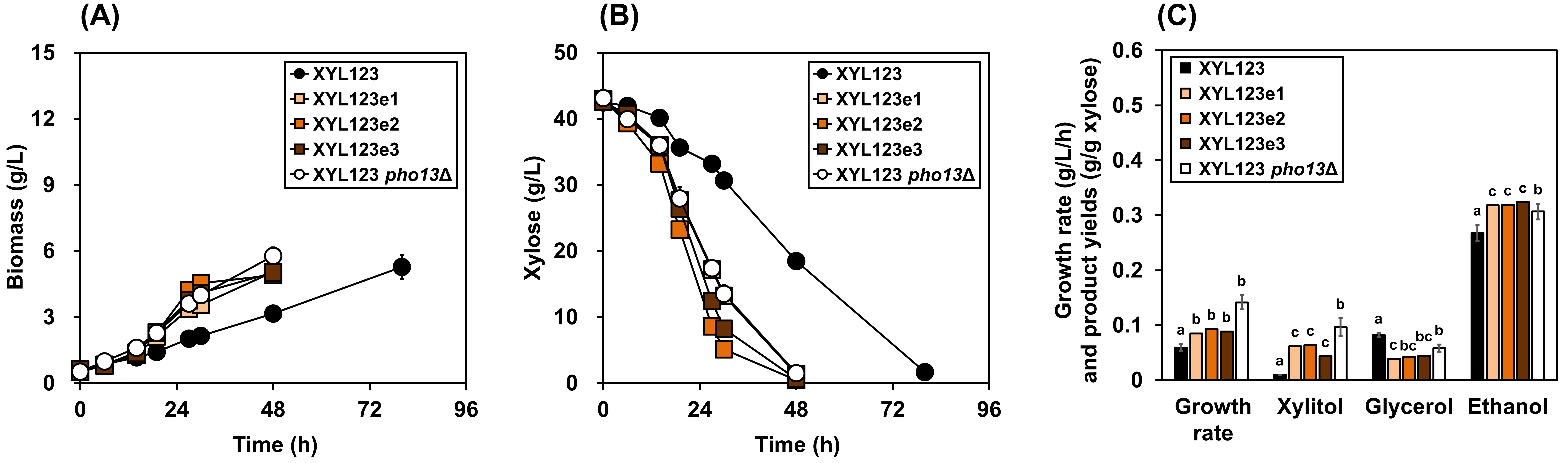

Supplement: S2 Fig — The XYL123 and XYL123 pho13Δ strains were used as the controls. Cell density (A), xylose concentrations (B), and fermentation parameters (C) were compared. Fermentations were performed in YP medium containing 40 g/L xylose under oxygen-limited conditions (80 rpm) with a starting OD600 of 1.0. Different letters (a, b, and c) represent significant differences (p < 0.05, ANOVA method). n. d.; Not detected. (TIF) [file pone.0236294.s003.tif]

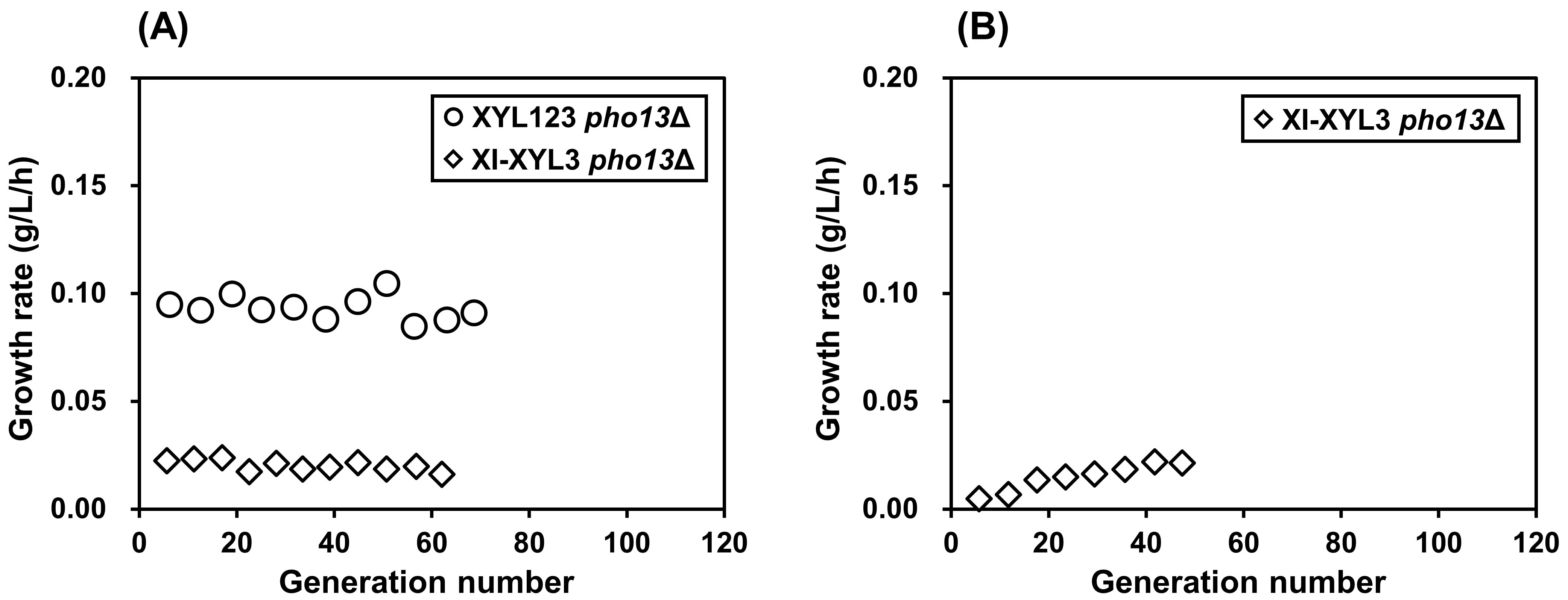

Supplement: S3 Fig — Under growth-liming concentrations of xylose, 40 g/L (A) and 100 g/L (A), the strains were serially subcultured until the described generation numbers. (TIF) [file pone.0236294.s004.tif]

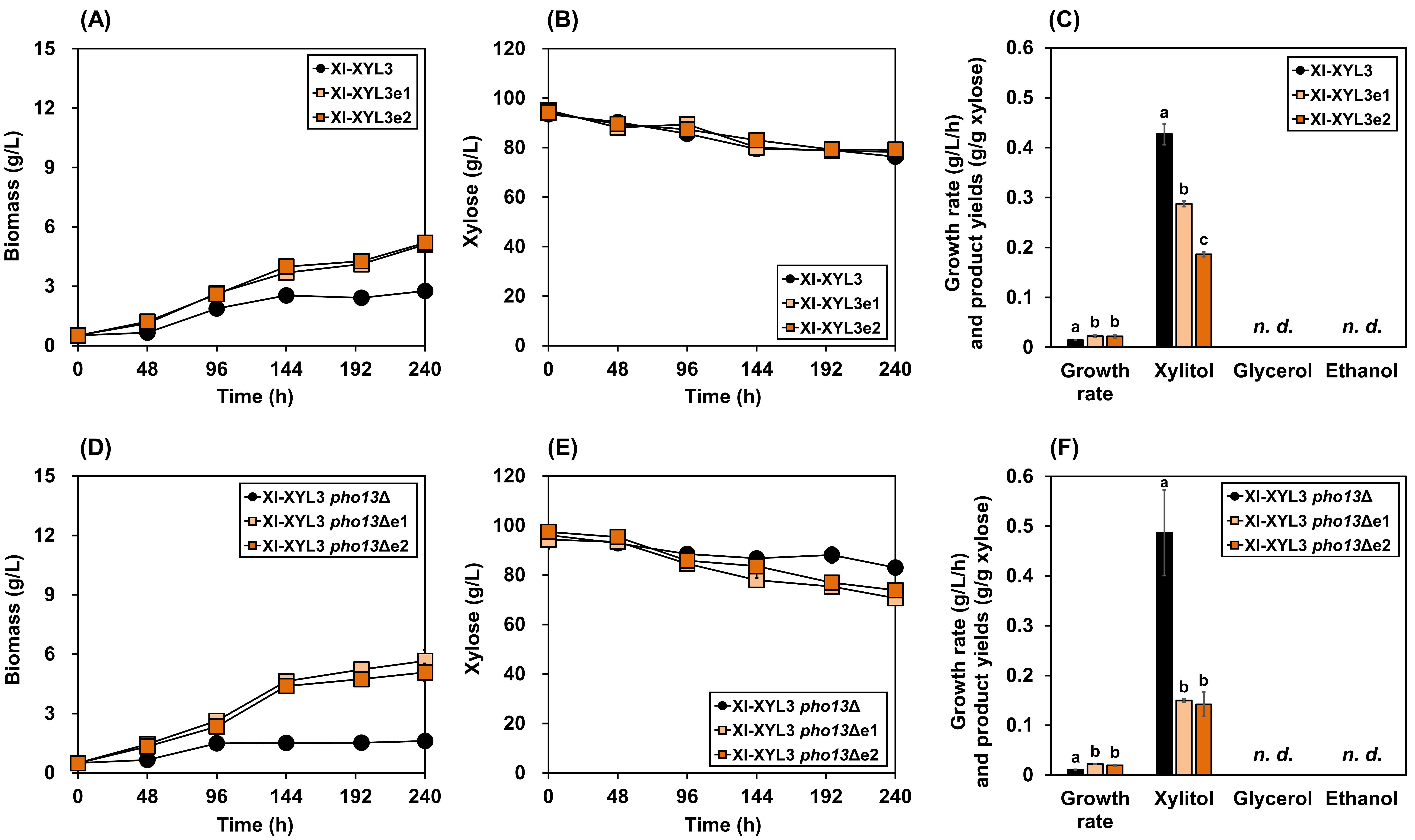

Supplement: S4 Fig — (A, B, C) The XI-XYL3 strain and its evolved strains (XI-XYL3e1, XI-XYL3e2). (C, D, E) The XI-XYL3 pho13Δ strain and its evolved strains (XI-XYL3 pho13Δe1, XI-XYL3 pho13Δe2). The strains were evaluated in YP medium containing 40 g/L xylose under oxygen-limited conditions (80 rpm) with a starting OD600 of 1.0. Different letters (a, b, and c) represent significant differences (p < 0.05, ANOVA method). n. d.; Not detected. (TIF) [file pone.0236294.s005.tif]

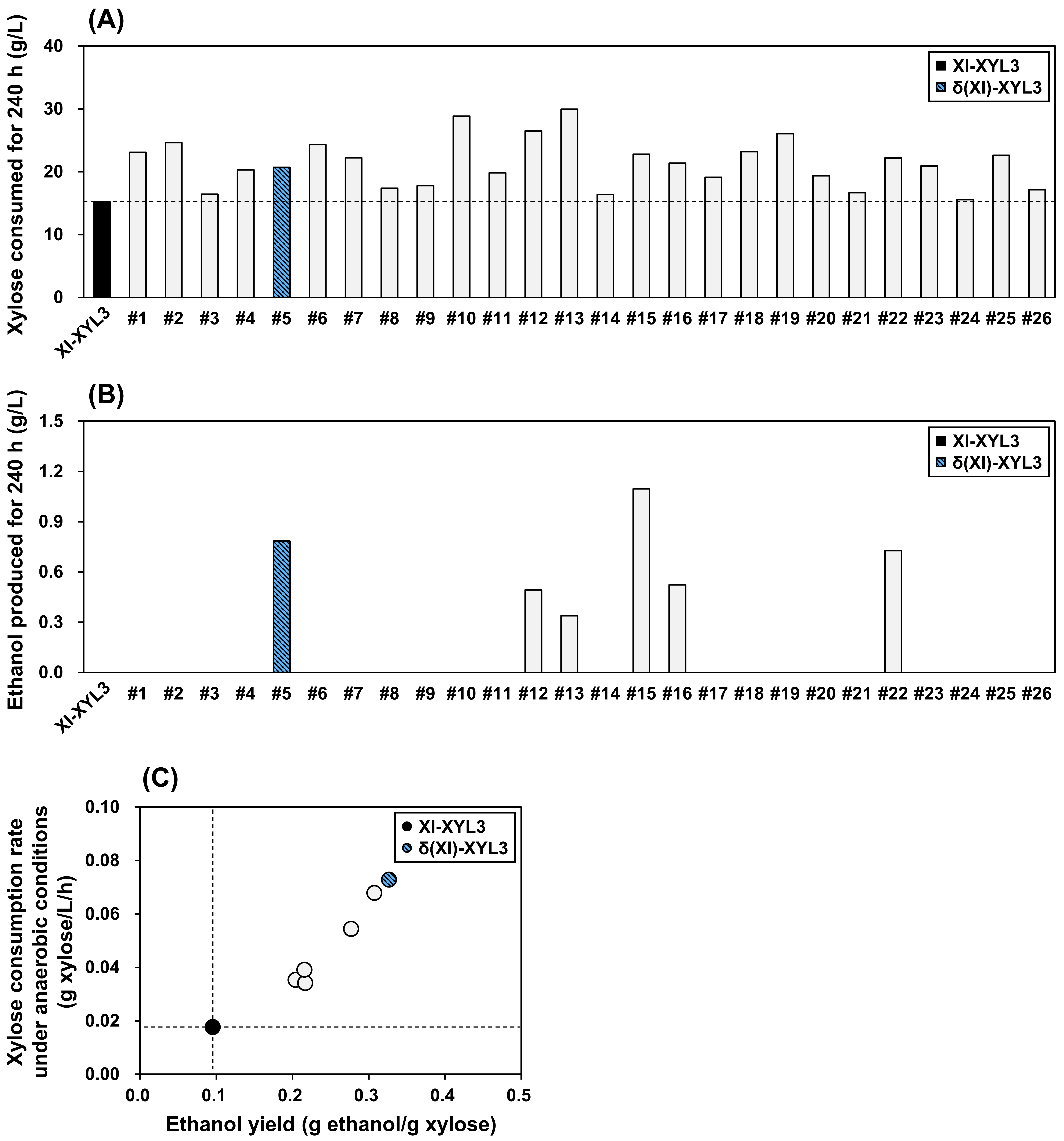

Supplement: S5 Fig — The XI-XYL3 strain and 26 mutants were evaluated the consumed xylose (g/L) (A) and the produced ethanol (g/L) (B) under oxygen-limited conditions (80 rpm). Six-mutants, which can produce ethanol, were selected and evaluated the xylose consumption rate (g xylose/L/h) and ethanol yield (g ethanol/g xylose) under oxygen-limited conditions (C) and anaerobic conditions (D). Fermentations were performed in YP medium containing 40 g/L xylose, with a starting OD600 of 1.0. The dashed line refer to the XI-XYL3 strain. (TIF) [file pone.0236294.s006.tif]

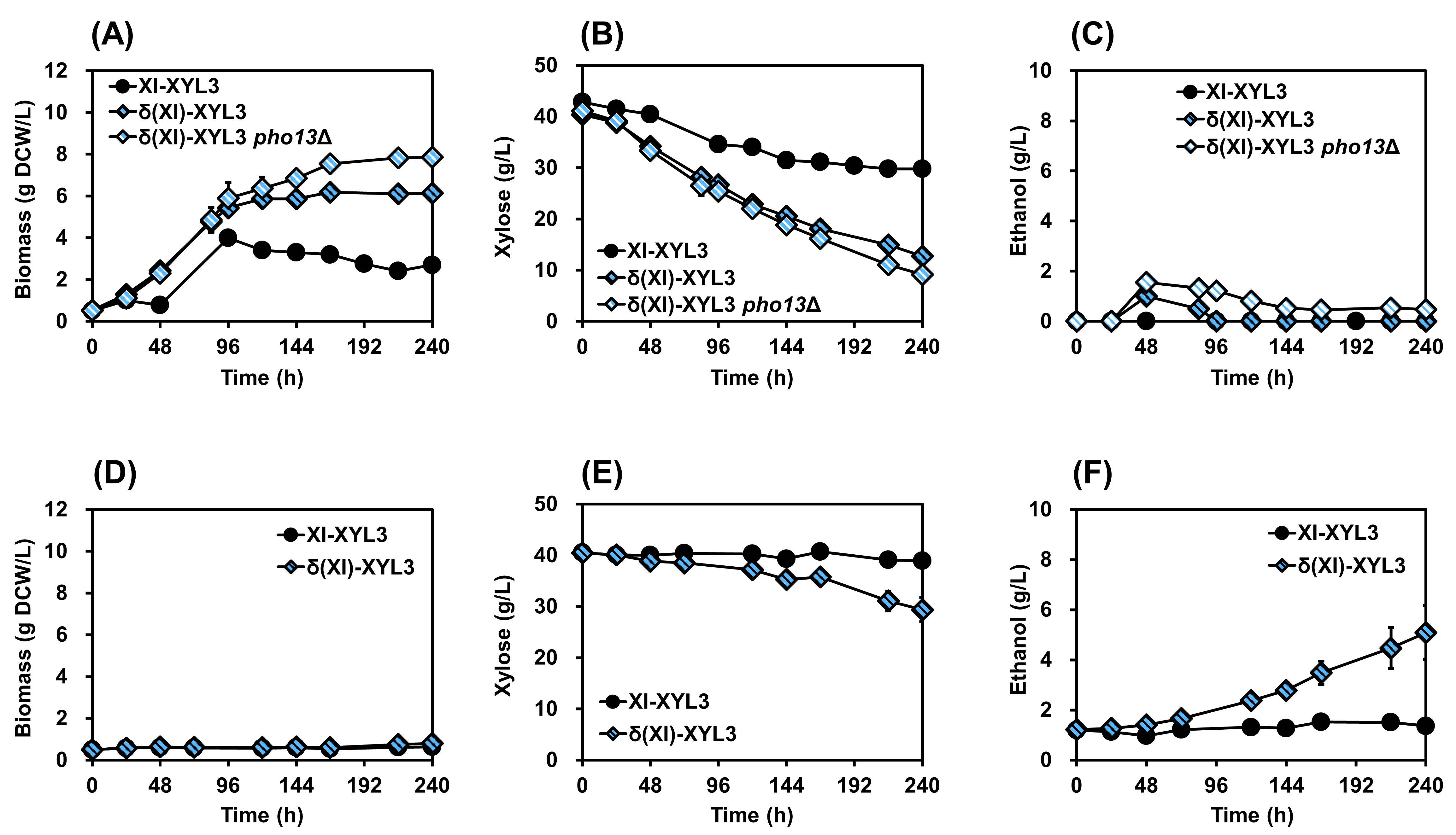

Supplement: S6 Fig — The strains were evaluated in YP medium containing 40 g/L xylose under oxygen-limited conditions (80 rpm, A-C) and anaerobic condition (D-F) with a starting OD600 of 1.0. (TIF) [file pone.0236294.s007.tif]

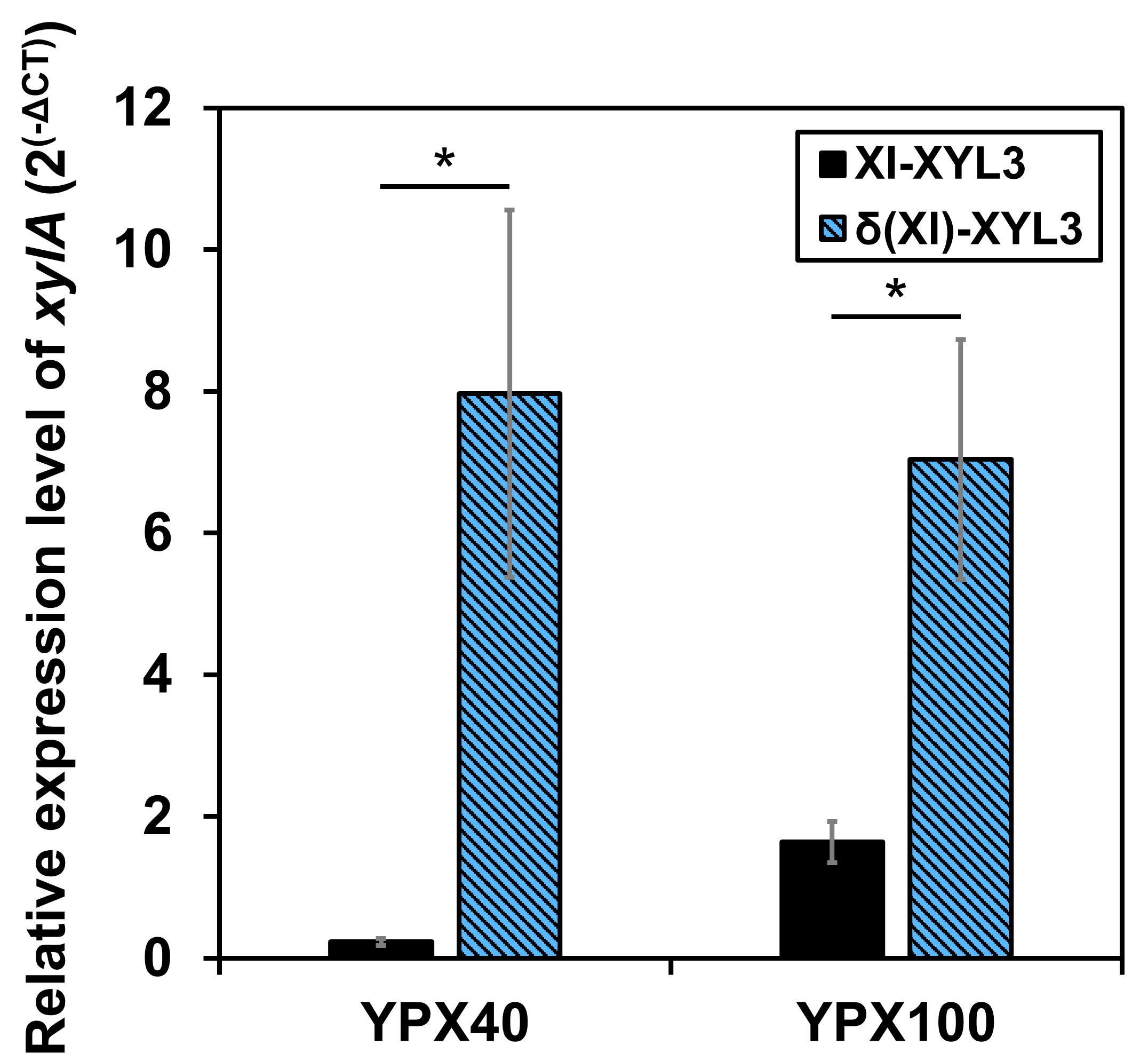

Supplement: S7 Fig — Increased transcriptional levels of the xylA gene in the XI-XYL3 and the xylA overexpressed strain (δ(XI)-XYL3) by δ-integration was confirmed by RT-qPCR. Fermentations were performed in YP medium containing 40 g/L (YPX40) or 100 g/L (YPX100) xylose, with a starting OD600 of 1.0. Asterisks denote statistically significant differences (Student's t-test, p < 0.05). (TIF) [file pone.0236294.s008.tif]
