# Supplementary material for: Metabolic engineering considerations for the heterologous expression of xylose-catabolic pathways in Saccharomyces cerevisiae
Source: PLoS One. 2020 Jul 27;15(7):e0236294. doi: 10.1371/journal.pone.0236294 (PMC7384654; doi:10.1371/journal.pone.0236294)
Supplement: S1 Table — (DOCX) [file pone.0236294.s009.docx]

**S1 Table. Plasmids used in this study**

| **Plasmids** | **Description/relevant genotype** | **Reference** |
| --- | --- | --- |
| pSR6-X123 | pRS306 *TDH3*_P_*-XYL1-TDH3*_T_-*PGK1*_P_*-XYL2-PGK1*_T_*-TDH3*_P_*-XYL3-TDH3*_T_ | [[1](#_ENREF_1)] |
| pRS42H-XI | pRS42H *TDH3*_P_*-xylA-TDH3*_T_ | This study |
| pYS10 | pRS305 *TDH3*_P_*-XYL1-TDH3*_T_ | [[2](#_ENREF_2)] |
| pYS-δXI | pRS305 *TDH3*_P_*-xylA-TDH3*_T_ | This study |
| pRS41N-Cas9 | A single-copy plasmid with a *natNT* marker and Cas9 | [[3](#_ENREF_3)] |
| pRS42K | A multi-copy plasmid with a *kanMX* marker | [[4](#_ENREF_4)] |
| pRS42H | A multi-copy plasmid with a *hphNT* marker | [[5](#_ENREF_5)] |
| pRS42H-ALD6.1 | pRS42H plasmid with a gRNA block containing 20-bp *ALD6* sequence (ALD6.1) | [[6](#_ENREF_6)] |
| pRS42H-PHO13.1 | pRS42H plasmid with a gRNA block containing 20-bp *PHO13* sequence (PHO13.1) | This study |
| pRS42K-PHO13.1 | pRS42K plasmid with a gRNA block containing 20-bp *PHO13* sequence (PHO13.1) | [[6](#_ENREF_6)] |
| pRS42H-TAL1.1 | pRS42H plasmid with a gRNA block containing 20-bp near *TAL1* sequence (TAL1.1) | [[7](#_ENREF_7)] |
| pRS42H-SOR1.1 | pRS42H plasmid with a gRNA block containing 20-bp *SOR1* sequence (SOR1.1) | [[6](#_ENREF_6)] |
| pRS42H-INT#1 | pRS42H plasmid with a gRNA block containing 20-bp near *HIP1* sequence (INT#1) | [[6](#_ENREF_6)] |
| pRS42H-INT#6 | pRS42H plasmid with a gRNA block containing 20-bp near *ATG33* sequence (INT#6) | [[8](#_ENREF_8)] |
| pRS42H-INT#9 | pRS42H plasmid with a gRNA block containing 20-bp near *GPH1* sequence (INT#9) | This study |

**References**

1. Kim SR, Ha S-J, Kong II, Jin Y-S (2012) High expression of *XYL2* coding for xylitol dehydrogenase is necessary for efficient xylose fermentation by engineered *Saccharomyces cerevisiae*. Metabolic Engineering 14: 336-343.

2. Jin Y-S, Jeffries TW (2003) Changing flux of xylose metabolites by altering expression of xylose reductase and xylitol dehydrogenase in recombinant *Saccharomyces cerevisiae*. Biotechnology for Fuels and Chemicals: Springer. pp. 277-285.

3. Kim SR, Xu H, Lesmana A, Kuzmanovic U, Au M, et al. (2015) Deletion of *PHO13*, encoding haloacid dehalogenase type IIA phosphatase, results in upregulation of the pentose phosphate pathway in *Saccharomyces cerevisiae*. Applied and Environmental Microbiology 81: 1601-1609.

4. Taxis C, Knop M (2006) System of centromeric, episomal, and integrative vectors based on drug resistance markers for *Saccharomyces cerevisiae*. Biotechniques 40: 73.

5. Zhang G-C, Kong II, Kim H, Liu J-J, Cate JH, et al. (2014) Construction of a quadruple auxotrophic mutant of an industrial polyploid *Saccharomyces cerevisiae* strain by using RNA-guided Cas9 nuclease. Applied and Environmental Microbiology 80: 7694-7701.

6. Ye S, Jeong D, Shon JC, Liu K-H, Kim KH, et al. (2019) Deletion of *PHO13* improves aerobic l-arabinose fermentation in engineered *Saccharomyces cerevisiae*. Journal of Industrial Microbiology & Biotechnology 46: 1725-1731.

7. Xu H, Kim S, Sorek H, Lee Y, Jeong D, et al. (2016) *PHO13* deletion-induced transcriptional activation prevents sedoheptulose accumulation during xylose metabolism in engineered *Saccharomyces cerevisiae*. Metabolic Engineering 34: 88-96.

8. Jeong D, Ye S, Park H, Kim SR (2020) Simultaneous fermentation of galacturonic acid and five-carbon sugars by engineered *Saccharomyces cerevisiae*. Bioresource Technology 295: 122259.
