# Supplementary material for: Metabolic engineering considerations for the heterologous expression of xylose-catabolic pathways in Saccharomyces cerevisiae
Source: PLoS One. 2020 Jul 27;15(7):e0236294. doi: 10.1371/journal.pone.0236294 (PMC7384654; doi:10.1371/journal.pone.0236294)
Supplement: S2 Table — (DOCX) [file pone.0236294.s010.docx]

**S2 Table. Primers and guide RNAs used in this study. Some primers were described in our prior studies [**[**1**](#_ENREF_1)**,**[**2**](#_ENREF_2)**]**

| **Primers** | **Sequences (5’-)** | **Description^a^** |
| --- | --- | --- |
| Kim044 | AAGATCACACTTCCAAAGTTTTAGAGCTAGAAATAGCAAG | FC_ALD6.1-F |
| Kim045 | TTGGAAGTGTGATCTTGACGATCATTTATCTTTCACTGCG | FC_ALD6.1-R |
| Kim624 | CTTATCTATTAACTTTCGTTTTAGAGCTAGAAATAGCAAG | FC_PHO13.1-F |
| Kim625 | AAAGTTAATAGATAAGGGAGATCATTTATCTTTCACTGCG | FC_PHO13.1-R |
| Kim444 | CGAGTATATAATTTTTCGTTTTAGAGCTAGAAATAGCAAG | FC_TAL1.1-F |
| Kim445 | AAAAATTATATACTCGAGAGATCATTTATCTTTCACTGCG | FC_TAL1.1-R |
| Kim236 | TTGGTGCAGGTCCTGTGGTTTTAGAGCTAGAAATAGCAAG | FC_SOR1.1-F |
| Kim237 | ACAGGACCTGCACCAAATAGATCATTTATCTTTCACTGCG | FC_SOR1.1-R |
| Kim141 | ACTTATCATTAAGAAAAGTTTTAGAGCTAGAAATAGCAAG | FC_int#1-F |
| Kim142 | TTTCTTAATGATAAGTATCGATCATTTATCTTTCACTGCG | FC_int#1-R |
| Kim314 | TCACAGTGTCACATCAGGTTTTAGAGCTAGAAATAGCAAG | FC_int#6-F |
| Kim315 | TGATGTGACACTGTGACAAGATCATTTATCTTTCACTGCG | FC_int#6-R |
| Kim498 | AAGAATACAACGCTCAAGTTTTAGAGCTAGAAATAGCAAG | FC_int#9_F |
| Kim499 | TGAGCGTTGTATTCTTCCCGATCATTTATCTTTCACTGCG | FC_int#9_R |
| Kim046 | TAACATACACAAACACATACTATCAGAATACAACCATGATTACGCCAAGC | Donor_ALD6.1-F |
| Kim047 | GTATATGACGGAAAGAAATGCAGGTTGGTACAAAGTTGGGTAACGCCAGG | Donor_ALD6.1-R |
| Kim068 | ATGTGACATCTTTACTATTCTCCAGCACGTTTACCATGATTACGCCAAGC | Donor_PHO13.2-F |
| Kim069 | CTATAACTCATTATTGGTTAAGGTGTAGATGAAGTTGGGTAACGCCAGG | Donor_PHO13.2-R |
| Kim785 | TCTCCAGCACGTTTTCAGTATTTACTTAATCGTATATTAAAGGAGCAATGC | Donor_PHO13.5-F |
| Kim786 | TTCAAAAAGTAATTCTACCCCTAGATTTTGCATTGCTCCTTTAATATACG | Donor_PHO13.5-R |
| SOO672 | CGGGTTTTTCTTTTTTCTCAATTCTTGGCTTCCTCTTGCTACCTATATTCCACCATAACA | Donor_TAL1.1-F |
| SOO673 | CAACCTTTTGTTTCTTTTGAGCTGGTTCAGACATGTTTAGTTAATTATAGTTCGTTGACC | Donor_TAL1.1-R |
| Kim238 | AATCAACAAGAAAAAATACTAAAAAAAAAAATTGAAAAATAAAGTGAATA | Donor_SOR1.1-F |
| Kim239 | TATATATGGACATGAACCAGTGCCGAAAAGTATTCACTTTATTTTTCAAT | Donor_SOR1.1-R |
| Kim129 | AGAAGTTTTTTTACCCCTCTCCACAGATCCAGGAAACAGCTATGACCATG | Donor_int#1-F |
| Kim130 | GACCGGGTAGATTTTTCCGTAACCTTGGTGTCTGTAAAACGACGGCCAGT | Donor_int#1-R |
| Kim320 | AGGAGACCGCTATAACCGGTTTGAATTTACAGGAAACAGCTATGACCATG | Donor_int#6.1-F |
| Kim321 | ATGAACTTGCTTGCTGTCAAACTTCTGAGTTGTGTAAAACGACGGCCAGT | Donor_int#6.1-R |
| Kim500 | GTAGCGGCATCGCCTAGTAGCACTGTGGGCAGGAAACAGCTATGACCATG | Donor_int#9.1-F |
| Kim501 | GGTGCTGGCTGCATCTCGCAGAGCTTCCTCTTTGTAAAACGACGGCCAGT | Donor_int#9.1-R |
| Kim262 | GAATTCGTAGACGCAGATAC | GRE3 seq_F |
| Kim263 | GCTATCATTGCTGTTTGACG | GRE3 seq_R |
| Kim049 | GGAACGGTGAGTGCAACG | ALD6_P1 |
| Kim078 | GATTGGAATTGGTTCGCAGTG | ALD6_P2 |
| Kim048 | GAGGAAGACGTTGAAGGTGG | PHO13_P1 |
| Kim077 | TTGGAGTTCAAACTGGCGAG | PHO13_P2 |
| SOO626 | TTTCTCAATTCTTGGCTTCCTC | Conf_TAL1p.1_F |
| SOO676 | AGACAATCTAGCATCAACTTCG | Conf_TAL1.1_R |
| Kim242 | CTCCGGTCTCGTATCTCCTTTC | SOR1_P1 |
| Kim243 | ATCTCGAATGAGCAGTTCTGG | SOR1_P2 |
| Kim143 | CATTATACGCACCCTAAGGGAC | int1_P1 |
| Kim144 | GTGAGTTCTCATAACCTCG | int1_P2 |
| Kim326 | GGTTCTGACTCCTACTGAGC | INT#6_P1 |
| Kim327 | AGCATCGAGTACGGCAGTTC | INT#6_P2 |
| Kim502 | TCCGACTAGCAAGGCAAC | INT#9_P1 |
| Kim503 | ATTCGTCGGTGTTCCTTCC | INT#9_P2 |
| Kim102 | GCCTTCTACGTTTCCATCCA | qPCR_ACT1_F |
| Kim103 | GGCCAAATCGATTCTCAAAA | qPCR_ACT1_R |
| Kim120 | CCTCAAGACTCCACAACTAACC | qPCR_TAL1.1_F |
| Kim121 | GGTGGTCTTACCATGCTTCTT | qPCR_TAL1.1_R |
| Kim018 | CGGAGGCCTTTAAGTTGGGTAACGCCAGG | M13fwd-T7 |
| Kim509 | CGGAAGCTTTGACCATGATTACGCCAAGC | M13rev-T3_HindIII |
| **Gene** | **guide RNA sequences(5’-)** | **References** |
| *ALD6* | GTCAAGATCACACTTCCAAA tgg | [[1](#_ENREF_1),[2](#_ENREF_2)] |
| *PHO13* | TCCCTTATCTATTAACTTTC cgg | [[1](#_ENREF_1),[2](#_ENREF_2)] |
| *TAL1* | TCTCGAGTATATAATTTTTC agg | [[3](#_ENREF_3)] |
| *SOR1* | TATTTGGTGCAGGTCCTGTG ggg | [[1](#_ENREF_1),[2](#_ENREF_2)] |
| *INT#1* | GATACTTATCATTAAGAAAA tgg | [[1](#_ENREF_1),[2](#_ENREF_2)] |
| *INT#6* | TTGTCACAGTGTCACATCAG cgg | [[4](#_ENREF_4)] |
| *INT#9* | GGGAAGAATACAACGCTCAA cgg | [[5](#_ENREF_5)] |

^a^ F, forward; R, reverse.

**References**

1. Jeong D, Ye S, Park H, Kim SR (2020) Simultaneous fermentation of galacturonic acid and five-carbon sugars by engineered *Saccharomyces cerevisiae*. Bioresource Technology 295: 122259.

2. Ye S, Jeong D, Shon JC, Liu K-H, Kim KH, et al. (2019) Deletion of *PHO13* improves aerobic l-arabinose fermentation in engineered *Saccharomyces cerevisiae*. Journal of Industrial Microbiology & Biotechnology 46: 1725-1731.

3. Xu H, Kim S, Sorek H, Lee Y, Jeong D, et al. (2016) *PHO13* deletion-induced transcriptional activation prevents sedoheptulose accumulation during xylose metabolism in engineered *Saccharomyces cerevisiae*. Metabolic Engineering 34: 88-96.

4. Ronda C, Maury J, Jakociunas T, Jacobsen SA, Germann SM, et al. (2015) CrEdit: CRISPR mediated multi-loci gene integration in *Saccharomyces cerevisiae*. Microbial cell factories 14: 97.

5. Sasano Y, Nagasawa K, Kaboli S, Sugiyama M, Harashima S (2016) CRISPR-PCS: a powerful new approach to inducing multiple chromosome splitting in *Saccharomyces cerevisiae*. Scientific Reports 6: 30278.
