# Supplementary material for: Metabolic engineering considerations for the heterologous expression of xylose-catabolic pathways in Saccharomyces cerevisiae
Source: PLoS One. 2020 Jul 27;15(7):e0236294. doi: 10.1371/journal.pone.0236294 (PMC7384654; doi:10.1371/journal.pone.0236294)
Supplement: S3 Table — (DOCX) [file pone.0236294.s011.docx]

**S3 Table.** **Fermentation profiles of evolved *S. cerevisiae* expressing the xylose oxidoreductase pathway**

| **Strain** | **Growth**  **rate**  **(g/L/h)** | **Xylose consumed**  **(g/L)** | **Xylose consumption rate (g/L/h)** | **Product titers (g/L)** | | | ***Y*_Xylitol_** | ***Y*_Glycerol_** | ***Y*_Ethanol_** | ***P*_Xylitol_^*^** | ***P*_Ethanol_^*^** |
| --- | --- | --- | --- | --- | --- | --- | --- | --- | --- | --- | --- |
|  |  |  |  | **Xylitol** | **Glycerol** | **Ethanol** |  |  |  |  |  |
| XYL123 | 0.06 ± 0.00 | 39.9 ± 1.0 | 0.53 ± 0.02 | 0.6 ± 0.2 | 3.3 ± 0.1 | 10.6 ± 0.4 | 0.01 ± 0.00 | 0.09 ± 0.01 | 0.26 ± 0.01 | < 0.00 | 0.06 ± 0.01 |
| XYL123 *pho13*∆ | 0.10 ± 0.01 | 41.1 ± 1.4 | 0.93 ± 0.04 | 3.7 ± 0.2 | 1.6 ± 0.4 | 11.9 ± 0.5 | 0.09 ± 0.01 | 0.04 ± 0.01 | 0.29 ± 0.01 | 0.03 ± 0.01 | 0.10 ± 0.01 |
| XYL123e1 | 0.17 | 41.5 | 0.86 | 2.6 | 1.6 | 13.2 | 0.06 | 0.04 | 0.32 | 0.02 | 0.12 |
| XYL123e2 | 0.19 | 42.5 | 0.89 | 2.7 | 1.8 | 13.6 | 0.06 | 0.04 | 0.32 | 0.02 | 0.11 |
| XYL123e3 | 0.18 | 41.9 | 0.87 | 1.8 | 1.9 | 13.6 | 0.04 | 0.04 | 0.32 | 0.01 | 0.11 |
| XI-XYL3 | 0.03 ± 0.00 | 17.1 ± 0.7 | 0.07 ± 0.00 | 7.3 ± 0.2 | *n. d.* | *n. d.* | 0.43 ± 0.02 | *n. d.* | *n. d.* | 0.02 ± 0.00 | *n. d.* |
| XI-XYL3e1 | 0.04 ± 0.00 | 16.9 ± 0.5 | 0.07 ± 0.00 | 4.9 ± 0.0 | *n. d.* | *n. d.* | 0.29 ± 0.01 | *n. d.* | *n. d.* | 0.01 ± 0.00 | *n. d.* |
| XI-XYL3e2 | 0.04 ± 0.01 | 14.9 ± 0.4 | 0.06 ± 0.00 | 2.8 ± 0.1 | *n. d.* | *n. d.* | 0.19 ± 0.00 | *n. d.* | *n. d.* | < 0.00 | *n. d.* |
| XI-XYL3 *pho13*Δ | 0.02 ± 0.00 | 13.1 ± 1.7 | 0.04 ± 0.01 | 6.3 ± 0.2 | *n. d.* | *n. d.* | 0.49 ± 0.09 | *n. d.* | *n. d.* | 0.02 ± 0.00 | *n. d.* |
| XI-XYL3 *pho13*Δe1 | 0.04 ± 0.00 | 23.7 ± 1.4 | 0.10 ± 0.02 | 3.5 ± 0.1 | *n. d.* | *n. d.* | 0.15 ± 0.00 | *n. d.* | *n. d.* | < 0.00 | *n. d.* |
| XI-XYL3 *pho13*Δe2 | 0.04 ± 0.00 | 23.6 ± 2.2 | 0.10 ± 0.01 | 3.3 ± 0.3 | *n. d.* | *n. d.* | 0.14 ± 0.02 | *n. d.* | *n. d.* | < 0.00 | *n. d.* |
| XI-XYL3 | 0.06 ± 0.00 | 18.8 ± 1.4 | 0.08 ± 0.01 | 5.8 ± 0.3 | *n. d.* | *n. d.* | 0.31 ± 0.03 | *n. d.* | *n. d.* | 0.01 ± 0.00 | *n. d.* |
| XI-XYL3 *TAL1* | 0.06 ± 0.00 | 16.8 ± 1.4 | 0.07 ± 0.00 | 5.1 ± 0.1 | *n. d.* | *n. d.* | 0.30 ± 0.01 | *n. d.* | *n. d.* | 0.01 ± 0.00 | *n. d.* |
| XI-XYL3 | 0.04 ± 0.01 | 14.7 ± 0.5 | 0.06 ± 0.00 | 5.0 ± 0.3 | *n. d.* | *n. d.* | 0.34 ± 0.01 | *n. d.* | *n. d.* | 0.01 ± 0.00 | *n. d.* |
| XI-XYL3 *gre3*Δ | 0.05 ± 0.00 | 15.4 ± 0.5 | 0.07 ± 0.01 | 4.9 ± 0.2 | *n. d.* | *n. d.* | 0.32 ± 0.00 | *n. d.* | *n. d.* | 0.01 ± 0.00 | *n. d.* |
| XI-XYL3 *sor1*Δ | 0.06 ± 0.00 | 17.4 ± 1.1 | 0.08 ± 0.01 | 5.1 ± 0.3 | *n. d.* | *n. d.* | 0.29 ± 0.00 | *n. d.* | *n. d.* | 0.01 ± 0.00 | *n. d.* |
| XI-(XYL3)_2_ | 0.05 ± 0.00 | 16.2 ± 1.2 | 0.07 ± 0.00 | 5.0 ± 0.4 | *n. d.* | *n. d.* | 0.30 ± 0.01 | *n. d.* | *n. d.* | 0.01 ± 0.00 | *n. d.* |

All strains were cultured in YP medium containing 40 g/L xylose under oxygen-limited conditions (80 rpm) with a low initial cell density (0.5 g DCW/L). All parameters were calculated when more than 90% of xylose was consumed. Acetate was not detected during the xylose fermentation.

Parameters: *Y*_Xylitol_, Xylitol yield (g xylitol/g xylose); *Y*_Glycerol_, Glycerol yield (g glycerol/g xylose); *Y*_Ethanol_, Ethanol yield (g ethanol/g xylose); *P*_Xylitol_*, Specific xylitol productivity (g/g cell/h); *P*_Ethanol_*, Specific ethanol productivity (g/g cell/h); *n. d.*, not detected.
